# Effects of diet type on the core fecal bacterial taxa and the dysbiosis index of healthy adult dogs

**DOI:** 10.3389/fvets.2025.1572875

**Published:** 2025-06-30

**Authors:** Patrícia M. Oba, Leah J. Roberts, Elizabeth L. Geary, Jan S. Suchodolski, Kelly S. Swanson

**Affiliations:** ^1^Department of Animal Sciences, University of Illinois Urbana-Champaign, Urbana, IL, United States; ^2^Gastrointestinal Laboratory, Department of Small Animal Clinical Sciences, Texas A&M University, College Station, TX, United States; ^3^Division of Nutritional Sciences, University of Illinois Urbana-Champaign, Urbana, IL, United States; ^4^Department of Veterinary Clinical Medicine, University of Illinois Urbana-Champaign, Urbana, IL, United States

**Keywords:** canine microbiome, canine nutrition, diet format, core microbiome, gastrointestinal microbiome

## Abstract

There is great interest in studying the canine gastrointestinal microbiome. In healthy dogs versus those with acute and chronic enteropathies, specific bacterial taxa have been identified that are consistently associated with shifts in the microbiome. A qPCR-based dysbiosis index (DI) that assesses microbiome shifts was developed based on a subset of these taxa. Because most dogs consume kibble diets, published data on core bacteria and the DI were largely derived from dogs consuming that diet form. Because dietary composition impacts the microbiome, it was unknown whether data from dogs consuming other diet types would adhere to reported core taxa abundance and DI guidelines. The study’s aim was to determine the fecal abundance of core bacteria and DI of dogs fed commercially available kibble vs. mildly-cooked human-grade (fresh) diets. Fecal samples collected from adult dogs across four experiments were used (4 kibble diets, *n* = 10–12/treatment; 4 fresh diets, *n* = 10–24/treatment). Moderate correlations were observed between total dietary fiber (TDF) and *Fusobacterium* (positive correlation), *Lactobacillus* (negative), and DI (negative). Dietary protein was correlated with fecal *Ruminococcus gnavus* (negative), while dietary fat was correlated with fecal *Bacteroides* and *C. perfringens* abundance (both positive). Dogs fed fresh diets exhibited higher (*p* < 0.01) abundances of *Streptococcus*, *Escherichia coli*, and *Clostridium perfringens*, while those fed kibble diets had higher (*p* < 0.05) abundances of *Fusobacterium*, *Clostridium hiranonis*, and *Bacteroides*. Dogs fed fresh diets had a greater (*p* < 0.0001) DI, but the majority of scores remained within the normal range. Dogs fed animal protein-based kibble diets had higher (*p* < 0.05) fecal *Faecalibacterium* and *Fusobacterium*, while dogs fed animal protein-based fresh diets had higher (*p* < 0.05) *Streptococcus*, *E. coli*, and *C. perfringens*. *Bifidobacterium* and *Bacteroides* were more abundant (*p* < 0.01) in dogs fed animal protein-based kibble and plant protein-based fresh diets. Dogs fed animal protein-based fresh diets had a greater (*p* < 0.0001) DI. Even though microbiota populations were statistically different among diets, all mean DI were <0, with only a few individual dogs consuming fresh diets having DI >0 (5 dogs >0; 1 dog >2). Overall, these data demonstrate the utility of the DI across different diet types in healthy dogs.

## 1 Introduction

Over the past decade, there has been significant interest in characterizing the gastrointestinal microbiome of dogs because of its role in digestion, energy extraction, pathogen defense, support for enterocytes, and immune stimulation (1–3). Most canine microbiome studies have focused on assessing the effects of various ingredients present in extruded kibble diets, which dominate the dog food market (4). The pet food industry frequently mirrors human dietary trends and has resulted in a great interest in the feeding of fresh diet formats. Unlike conventional extruded diets, fresh diets utilize methods like kettle cooking and steam cooking, with many cooking individual ingredients before blending. Conventional pet foods usually include animal and plant byproducts, but an emerging preference among pet owners is to choose byproduct-free diets (5). The macronutrient composition of these diets may differ based on these preferences or manufacturing method, with potential influences on the nutritional and health status of animals.

Dietary differences in manufacturing method, ingredient composition, and nutrient concentration may impact nutrient digestibility and consequent influences on the microbiome. Alpha and beta diversity results have been inconsistent across previous microbiome studies. For instance, dogs fed fresh diets have been shown to have lower species richness than those fed kibble diets (6). However, other studies found no differences in alpha diversity between dogs fed fresh diet and those fed kibble diets (7–9). Some studies have reported significant shifts in beta diversity when comparing fresh diets to kibble diets (8); however, other studies have not found significant separation between microbial communities (6, 7, 9). These findings underscore that diet type may affect gut microbiota composition, although there are major inconsistencies in the reported microbiome results across studies.

Eubiosis, or microbial ecosystem balance, is present in a healthy state, where the symbiotic relationship between the host and microorganisms maintains a state of equilibrium. Disturbances may lead to a state of dysbiosis, often characterized by reduced bacterial diversity, depletion of beneficial bacteria, and the proliferation of pathobionts, resulting in altered metabolic functionality (10, 11). Dysbiosis is implicated in various diseases such as inflammatory bowel disease (IBD), obesity, allergy, and diabetes in humans, rodent models, and companion animals (12). The collection of studies testing the gut microbiota populations of individuals with these conditions, however, have not described a consistent microbial shift. For instance, in dogs with chronic enteropathies, some have reported greater *Gammaproteobacteria* and reduced *Erysipelotrichia*, *Clostridia*, and *Bacteroidia* (13). Another study noted elevated *Sutterella* and *Clostridium perfringens* alongside reduced *Blautia*, Ruminococcaceae, and *Turicibacter* (14). Others reported decreases in bacterial groups within the phyla Firmicutes and Bacteroidetes, increases within Proteobacteria, and reduction in Lachnospiraceae and *Clostridium coccoides* subgroups (15). Despite these disparities across studies, they highlight that a reduction of *Clostridium* clusters XIVa and IV are associated with IBD in dogs (13–16). These results underscore the pivotal role of these bacterial clusters, which are known short-chain fatty acid (SCFA) producers, in maintaining gastrointestinal health (15).

A major challenge in interpreting results of microbiome data, which has recently been highlighted, is the lack of standardization and high inter-assay variability of next-generation sequencing (NGS) methods that are commonly used in microbiome studies (17, 18). This leads to lack of reproducibility and contributes to the variation reported across different studies when evaluating dietary or health effects. For example, even when the same samples are analyzed, the choices of sample preparation, sequencing protocols, and bioinformatics (i.e., databases, statistical tests) leads to significantly different reported results and interpretations between laboratories (17, 18). Next-generation sequencing data shows high assay variability (19), and due to the compositional data of results (i.e., data is reported as relative percentages), analytical-induced changes in some taxa per definition leads to changes in all other taxa due to method variation rather than because of biological variation (20). Furthermore, some important bacterial taxa (i.e., *Clostridium hiranonis*) remain undetected by NGS, while targeted quantitative PCR is able to detect these taxa with higher analytical sensitivity and reproducibility (21–23).

A dysbiosis index (DI) was developed by employing qPCR analysis of some core bacterial taxa, particularly in the context of healthy dogs vs. those with chronic enteropathies (24). The DI is used with clinical outcomes to aid in disease diagnosis and management, dividing samples into four groups. A DI <0 and with all targeted taxa within the reference interval are considered normal, a DI <0 but with any of the targeted taxa outside the reference interval are defined as having a minor shift in the microbiome, a DI between 0 and 2 is defined as a mild to moderate microbiome shift, and a DI >2 is considered to be significant dysbiosis. Development of the DI was largely based on samples from dogs consuming extruded kibble diets, as it is the most popular diet format among United States dog owners (25, 26). As stated above, the dietary composition (including ingredients and nutrients) and processing methods have an impact on nutrient digestibility and the fecal microbiome (6–9). Given the impact that diet has on the microbiome, it is important to evaluate whether the DI guidelines are appropriate for dogs fed non-kibble diets.

Furthermore, evaluating dietary induced changes in core bacterial taxa in dogs using the same reproducible method by quantitative PCR would allow better comparison across future studies (23, 27). Therefore, the primary aim of the current study was to compare the fecal core bacteria and DI of dogs fed commercially available fresh diets versus those fed extruded kibble diets. A secondary aim was to compare these core bacteria and DI of dogs fed fresh animal protein-based diets (FAP), fresh plant protein-based diets (FPB), and kibble animal protein-based diets (KAP).

## 2 Materials and methods

All procedures were approved by the University of Illinois Institutional Animal Care and Use Committee prior to experimentation and were performed in accordance with the U. S. Public Health Service Policy on Humane Care and Use of Laboratory Animals.

### 2.1 Experimental design

Fresh fecal samples from 37 adult spayed female Beagle dogs from previous studies (8, 9, 28) were used for the analyses. All dogs were adult spayed females in a healthy condition (between 3–7 years old) and were within a healthy body weight range at the time of sample collection. To be included in this experiment, dogs had not been administered any medications (antibiotics, antacids, anti-inflammatory medications, or corticosteroids) that could potentially impact their gut microbiota for a minimum of 4 weeks prior to and during the course of the study. Dogs were housed individually in pens (1.22 m wide × 1.85 m long) in a temperature-controlled room under a 12 h light: 12 h dark cycle at the University of Illinois Urbana-Champaign. As per the University of Illinois IACUC requirements, all dogs received a minimum of two socialization sessions per week with two trained personnel. Additionally, they were bathed every other week (every 14 days) and had continuous access to at least one enrichment toy in their run at all times. Dogs had free access to fresh water and were fed to maintain body weight throughout the studies. Dogs were fed once (28) or twice (8, 9) daily. Before collecting fresh fecal samples, dogs were acclimated to the diet for a minimum of 7 days.

### 2.2 Diets

All diets tested were commercially available and formulated to meet Association of American Feed Control Officials (63) nutrient profiles for adult dogs at maintenance. Test diets included those listed below (Table 1).

**Table 1:** Guaranteed analysis and ingredient list of diets.

Item	Kibble			Fresh			
	KAP^a^			FAP^a^		FPB^a^	
	Fit & Trim^b^	Orijen Original^c^	Chicken & Brown Rice^d^	Chicken Cuisine^e^	Chicken & White Rice^f^	Cowbell^g^	Roost^h^
Moisture (max.), %	12	12	10	77	72	65	65
Dry matter basis							
Crude protein (min.), %	48	43	27	37	29	31	36
Crude fat (min.), %	15	20	16	26	11	20	23
Crude fiber (max.), %	9	5	6	4	4	3	3

a KAP, kibble animal protein-based diets; FAP, fresh animal protein-based diets; FPB, fresh plant protein-based diets.

b Fit & Trim, Champion Petfoods LP, Edmonton, Canada. Ingredients: fresh chicken meat, fresh cage-free eggs, fresh whole herring, fresh turkey meat, fresh chicken liver, fresh whole flounder, fresh whole mackerel, fresh whole Pacific hake, fresh turkey liver, fresh chicken heart, chicken (dehydrated), turkey (dehydrated), whole mackerel (dehydrated), whole sardine (dehydrated), whole herring (dehydrated), Alaskan pollock (dehydrated), lentil fiber, whole red lentils, whole green lentils, fresh whole green peas, fresh whole chickpeas, fresh whole yellow peas, whole pinto beans, whole navy beans, chicken cartilage (dehydrated), fresh turkey heart, apple fiber, dried algae (source of DHA and EPA), pumpkin (dehydrated), butternut squash (dehydrated), carrots (dehydrated), chicken liver (freeze-dried), turkey liver (freeze-dried), fresh whole pumpkin, fresh whole butternut squash, fresh whole zucchini, fresh whole parsnips, fresh carrots, fresh whole Red Delicious apples, fresh whole Bartlett pears, fresh kale, fresh spinach, fresh beet greens, fresh turnip greens, brown kelp, whole cranberries, whole blueberries, whole Saskatoon berries, chicory root, turmeric root, milk thistle, burdock root, lavender, marshmallow root, rosehips.

c Orijen Original, Champion Petfoods LP, Edmonton, Canada. Ingredients: fresh chicken meat, fresh turkey meat, fresh cage-free eggs, fresh chicken liver, fresh whole herring, fresh whole flounder, fresh turkey liver, fresh chicken necks, fresh chicken heart, fresh turkey heart, chicken (dehydrated), turkey (dehydrated), whole mackerel (dehydrated), whole sardine (dehydrated), whole herring (dehydrated), whole red lentils, whole green lentils, whole green peas, lentil fiber, whole chickpeas, whole yellow peas, whole pinto beans, whole navy beans, herring oil, chicken fat, chicken cartilage, chicken liver (freeze-dried), turkey liver (freeze-dried), fresh whole pumpkin, fresh whole butternut squash, fresh whole zucchini, fresh whole parsnips, fresh carrots, fresh whole Red Delicious apples, fresh whole Bartlett pears, fresh kale, fresh spinach, fresh beet greens, fresh turnip greens, brown kelp, whole cranberries, whole blueberries, whole Saskatoon berries, chicory root, turmeric root, milk thistle, burdock root, lavender, marshmallow root, rosehips.

d Chicken & Brown Rice, Blue Buffalo, Wilton, CT. Ingredients: deboned chicken, chicken meal, brown rice, barley, oatmeal, pea starch, flaxseed, chicken fat, dried tomato pomace, natural flavor, peas, pea protein, salt, potassium chloride, dehydrated alfalfa meal, potatoes, dried chicory root, pea fiber, alfalfa nutrient concentrate, calcium carbonate, choline chloride, DL-methionine, preserved with mixed tocopherols, dicalcium phosphate, sweet potatoes, carrots, garlic, zinc amino acid chelate, zinc sulfate, vegetable juice for color, ferrous sulfate, vitamin E supplement, iron amino acid chelate, blueberries, cranberries, barley grass, parsley, turmeric, dried kelp, *Yucca schidigera* extract, niacin (vitamin B3), glucosamine hydrochloride, calcium pantothenate (vitamin B5), copper sulfate, biotin (vitamin B7), L-ascorbyl-2-polyphosphate, L-lysine, L-carnitine, vitamin A supplement, copper amino acid chelate, manganese sulfate, taurine, manganese amino acid chelate, thiamin mononitrate (vitamin B1), riboflavin (vitamin B2), vitamin D3 supplement, vitamin B12 supplement, pyridoxine hydrochloride (vitamin B6), calcium iodate, dried yeast, dried *Enterococcus faecium* fermentation extract, dried *Trichoderma longibrachiatum* fermentation extract, dried *Bacillus subtilis* fermentation extract, folic acid (vitamin B9), sodium selenite, oil of rosemary.

e Chicken Cuisine, NomNomNow, Inc., Nashville, TN. Ingredients: diced chicken, sweet potatoes, water sufficient for processing, squash, spinach, natural flavor, sunflower oil, canola oil, dicalcium phosphate, vinegar, citric acid, salt, calcium carbonate, fish oil, taurine, choline bitartrate, dimagnesium phosphate, zinc gluconate, iron amino acid chelate, vitamin E supplement, potassium iodide, vitamin B12 supplement, copper gluconate, manganese gluconate, selenium amino acid chelate, niacin (vitamin B3), thiamine mononitrate (vitamin B1), pyridoxine hydrochloride (vitamin B6), riboflavin (vitamin B2), folic acid, vitamin A supplement, cholecalciferol (source of vitamin D3), pantothenic acid (calcium-D-pantothenate).

f Chicken & White Rice, Just Food For Dogs, Irvine, CA. Ingredients: chicken thigh, long grain white rice, spinach, carrots, apples, chicken gizzard, chicken liver, eicosapentaenoic acid (EPA) and docosahexaenoic acid (DHA), dicalcium phosphate dihydrate, calcium, sodium chloride, choline bitartrate, dried seaweed meal, zinc oxide, magnesium amino acid chelate, vitamin E (as a-tocopherol succinate), ferrous amino acid chelate, copper amino acid chelate, vitamin D3 (as cholecalciferol), vitamin B5 (as calcium D-pantothenate), riboflavin, vitamin B12 (as cyanocobalamin).

g Cowbell, Bramble, Inc., New York, NY. Ingredients: organic pea protein, lentil, sweet potato, carrots, organic sunflower oil, organic flax oil, peas, apples, malt extract, potato starch, nutrient mix [choline chloride, potassium chloride, L-methionine, tricalcium phosphate, taurine], vitamins (D-calcium pantothenate, riboflavin, niacin, vitamin B12, vitamin A acetate, vitamin E supplement, folic acid, thiamin mononitrate, pyridoxine hydrochloride, vitamin D2 supplement), trace minerals (zinc proteinate, iron proteinate, copper proteinate, manganese proteinate, calcium iodate, selenium yeast), nutritional yeast, caramel color, tricalcium phosphate, potassium chloride, sodium phosphate, magnesium, salt.

h Roost, Bramble, Inc., New York, NY. Ingredients: organic pea protein, long grain brown rice, potato, garbanzo beans, carrots, organic sunflower oil, peas, butternut squash, blueberries, malt extract, potato starch, nutrient mix (choline chloride, potassium chloride, L-methionine, tricalcium phosphate, taurine), vitamins (D-calcium pantothenate, riboflavin, niacin, vitamin B12, vitamin A acetate, vitamin E supplement, folic acid, thiamin mononitrate, pyridoxine hydrochloride, vitamin D2 supplement), trace minerals (zinc proteinate, iron proteinate, copper proteinate, manganese proteinate, calcium iodate, selenium yeast), nutritional yeast, tricalcium phosphate, potassium chloride, sodium phosphate, magnesium, salt.

#### 2.2.1 KAP diets (*n* = 45 samples)

- Fit & Trim, Champion Petfoods LP, Edmonton, Canada.
- Orijen Original, Champion Petfoods LP, Edmonton, Canada.
- Chicken & Brown Rice, Blue Buffalo, Wilton, CT.

#### 2.2.2 FAP diets (*n* = 34 samples)

- Chicken & White Rice, Just Food For Dogs, Irvine, CA.
- Chicken Cuisine, NomNomNow, Inc., Nashville, TN.

#### 2.2.3 FPB diets (*n* = 22 samples)

- Cowbell, Bramble, Inc., New York, NY.
- Roost, Bramble, Inc., New York, NY.

### 2.3 Fecal sample collection

One fresh fecal sample from each dog was collected within 15 min of defecation and immediately transferred to sterile cryogenic vials (Nalgene, Rochester, NY). Cryovials were stored on dry ice until being transferred to a −80°C freezer. At a later date, samples were placed in dry ice and shipped to the Gastrointestinal Laboratory at Texas A&M University for analysis.

### 2.4 Quantitative PCR and dysbiosis index

DNA was extracted from an aliquot of 100–120 mg fecal sample using a bead-beating method with a MoBio Power soil DNA isolation kit. qPCR analysis of core bacterial taxa (23, 27) was performed with specific primers targeting *Faecalibacterium*, *Fusobacterium*, *Blautia*, universal bacteria, *Turicibacter*, *Escherichia coli*, *Clostridium hiranonis*, and *Streptococcus* as described in (24). In addition to the bacterial groups included in the DI calculation, *Bacteroides*, *Bifidobacterium*, *Clostridium perfringens*, *Collinsella*, *Lactobacillus*, *Prevotella copri*, and *Ruminococcus gnavus* were also quantified by qPCR as described before (23). Specific primers and PCR conditions are listed in Supplementary File 1. Briefly, the conditions for qPCR were as follows: initial denaturation at 98°C for 2 min, then 40 cycles with denaturation at 98°C for 3 s, and annealing for 3 s. Melt curve analysis was performed to validate the specific generation of the qPCR product using these conditions: 95°C for 1 min, 55°C for 1 min, and increasing incremental steps of 0.5°C for 80 cycles for 5 s each. Each reaction was run in duplicate. The qPCR data were expressed as the log amount of DNA (fg) for each particular bacterial group/10 ng of isolated total DNA as reported previously (14, 29). The degree of dysbiosis is represented as a single numerical value that measures the closeness of a taxa compared with the mean abundances derived from healthy and diseased populations and is calculated by an Euclidean distance model, as detailed previously (24). The reference intervals for each taxa and the dysbiosis index were based on the study conducted by AlShawaqfeh et al. (24).

### 2.5 Statistical analyses

The data underwent analysis through R (version 4.3.1, R Core Team). Given the lack of normality in the dataset, pairwise comparisons between the groups were conducted using Wilcoxon tests with Bonferroni correction for multiple testing from the ggpubr package (version 0.6.0). Correlation tests between nutrient concentrations, bacterial abundance, and the DI were performed using Spearman’s rank correlation from the stats package (version 4.3.3). Because the chemical composition of the NomNomNow diet was not analyzed, samples from that diet were excluded from the correlation analyses. Statistical significance was determined at a threshold of *p* < 0.05. Plot graphics were generated using ggplot2 package (version 3.5.1).

## 3 Results

**Figure 1:**
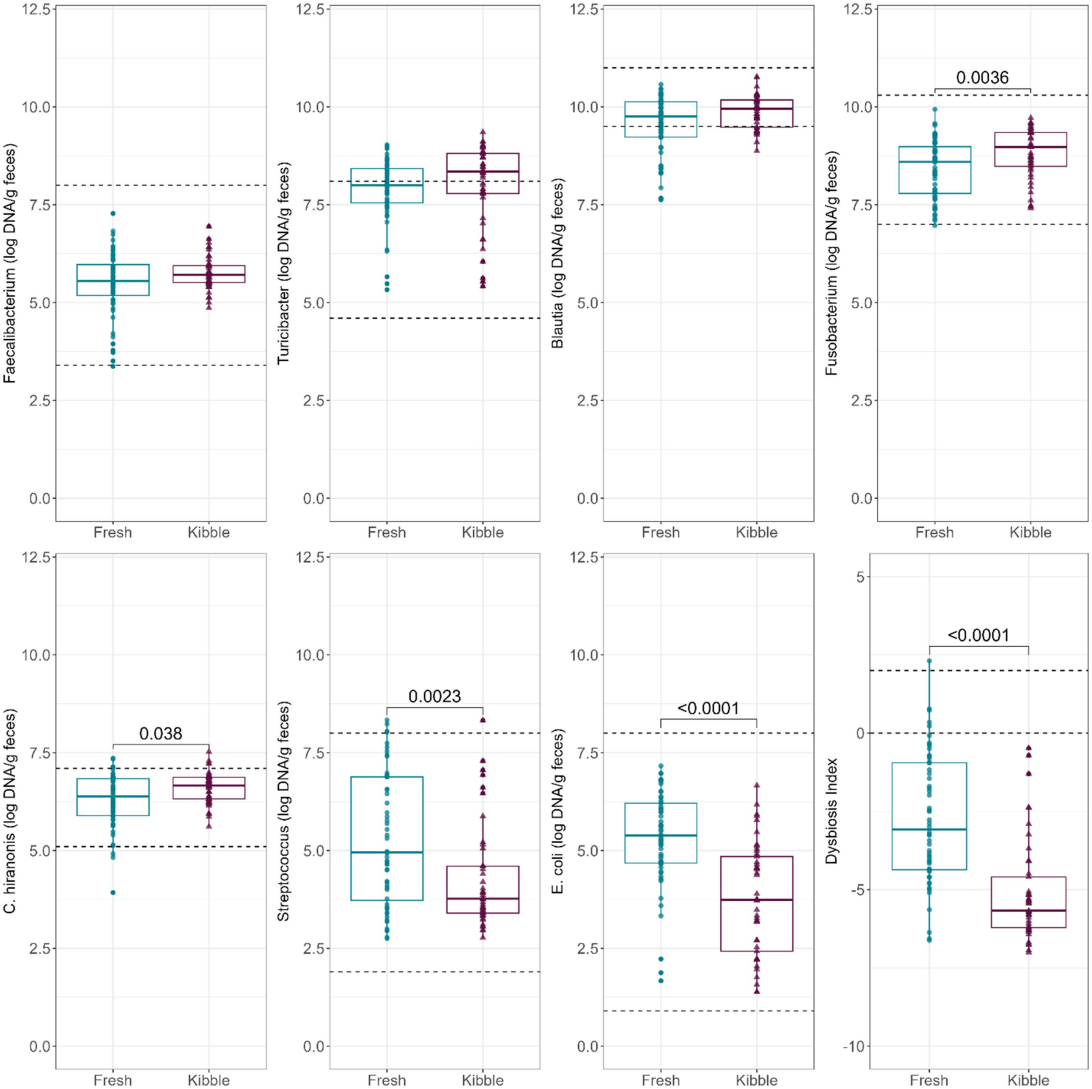
Abundances of fecal *Faecalibacterium*, *Turicibacter*, *Blautia*, *Fusobacterium*, *C. hiranonis*, *Streptococcus*, and *E. coli* (log DNA/g feces) and DI of dogs fed fresh or kibble diets. Horizontal lines are the normal reference ranges for each bacterial taxa. For DI, the horizontal lines indicate the thresholds for mild and severe dysbiosis.

Abundances of fecal *Fusobacterium*, *C. hiranonis*, *Streptococcus*, and *E. coli* (log DNA/g feces) and the DI were statistically impacted by diet type, although the majority of samples remained in the reported reference intervals (Figure 1). Fecal abundances of *Streptococcus* (*p* = 0.0023) and *E. coli* (*p* < 0.0001) were higher in dogs fed fresh diets than those fed kibble diets. In contrast, fecal abundances of *Fusobacterium* (*p* = 0.0036) and *C. hiranonis* (*p* = 0.0380) were higher in dogs fed kibble diets than those fed fresh diets. The DI was greater (*p* < 0.0001) in dogs fed fresh diets than those fed kibble diets. Despite the large differences in fecal microbiota, all mean DI were <0. A few individual dogs fed fresh diets had a DI >0 (5 dogs >0 and 1 dog >2).

**Figure 2:**
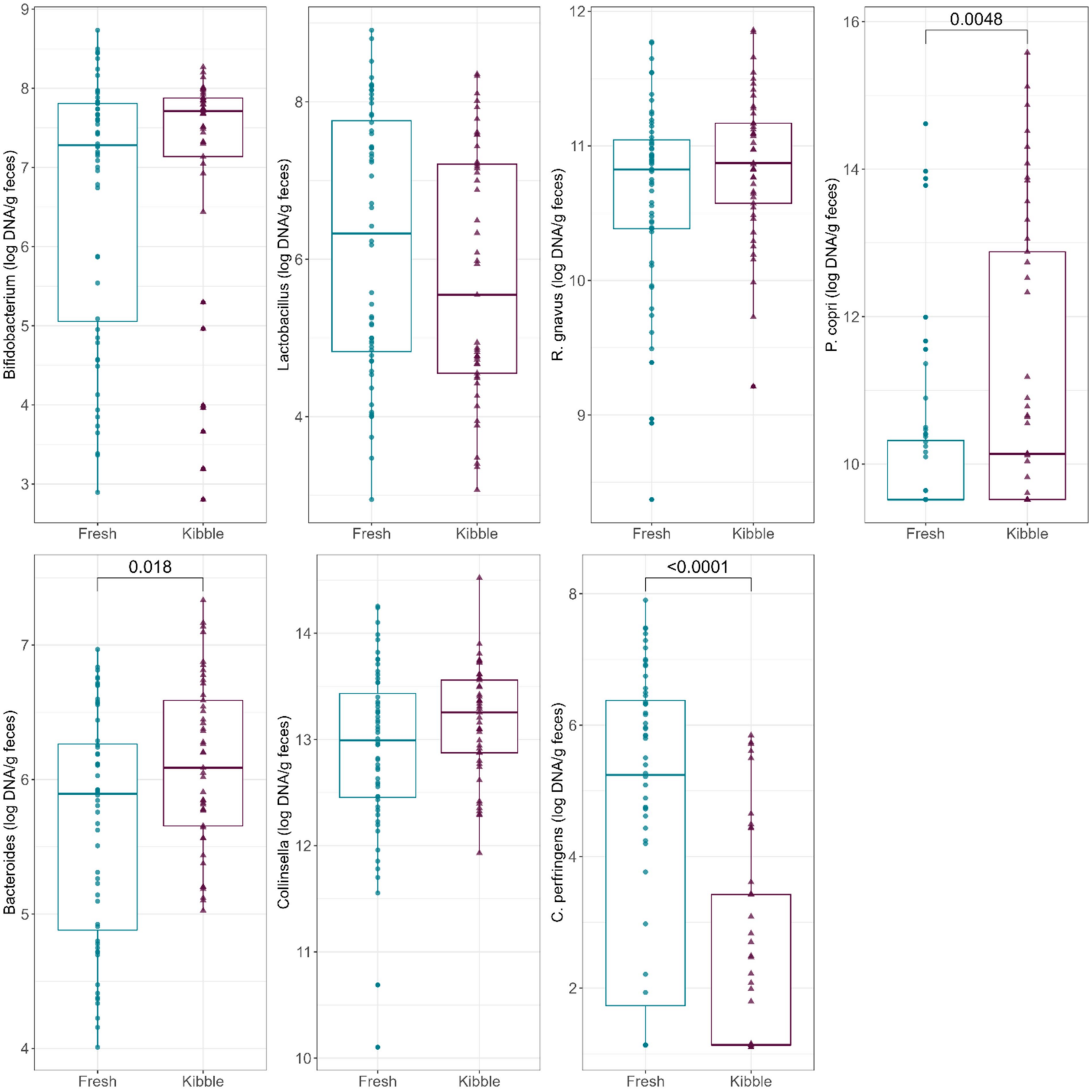
Abundances of fecal *Bifidobacterium*, *Lactobacillus*, *R. gnavus*, *P. copri*, *Bacteroides*, *Collinsella*, and *C. perfringens* (log DNA/g feces) of dogs fed fresh or kibble diets.

Abundances of fecal *Bacteroides* and *C. perfringens* were also impacted by diet type (Figure 2). Fecal *Bacteroides* abundance was higher (*p* = 0.0180) in dogs fed kibble diets than those fed fresh diets. Fecal *C. perfringens* abundance was higher (*p* < 0.0001) in dogs fed fresh diets than those fed kibble diets.

**Figure 3:**
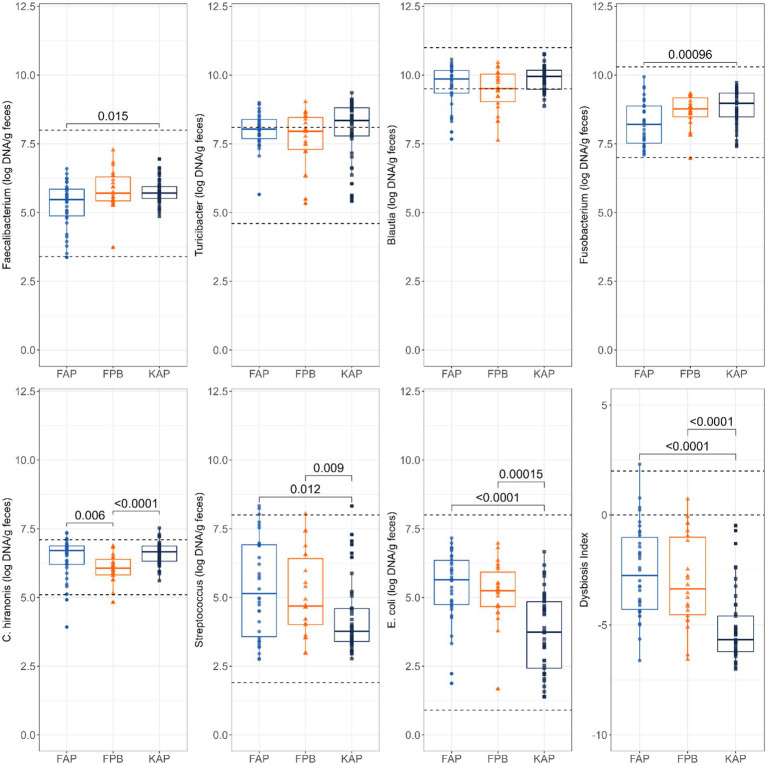
Abundances of fecal *Faecalibacterium*, *Turicibacter*, *Blautia*, *Fusobacterium*, *C. hiranonis*, *Streptococcus*, and *E. coli* (log DNA/g feces) and DI of dogs fed fresh animal protein-based (FAP), fresh plant protein-based (FPB), and kibble animal protein-based (KAP) diets. Horizontal lines are the normal reference ranges for each bacterial taxa. For DI, the horizontal lines indicate the thresholds for mild and severe dysbiosis.

Abundances of fecal *Faecalibacterium*, *Fusobacterium*, *C. hiranonis*, *Streptococcus*, and *E. coli* (log DNA/g feces) and the DI were impacted by protein source and diet type (Figure 3). Fecal abundances of *Faecalibacterium* (*p* = 0.015) and *Fusobacterium* (*p* = 0.0010) were higher in dogs fed KAP diets than those fed FAP diets. Fecal abundance of *C. hiranonis* was higher in dogs fed FAP diets (*p* = 0.006) and KAP diets (*p* < 0.0001) than dogs fed FPB diets. Fecal abundance of *Streptococcus* was higher in dogs fed FAP diets (*p* = 0.012) and FPB diets (*p* = 0.009) than dogs fed KAP diets. Likewise, fecal abundance of *E. coli* was higher in dogs fed FAP diets (*p* < 0.0001) and FPB diets (*p* = 0.0002) than those fed than those fed KAP diets. The DI was greater (*p* < 0.0001) in dogs fed the FAP and FPB diets than those fed the KAP diets.

**Figure 4:**
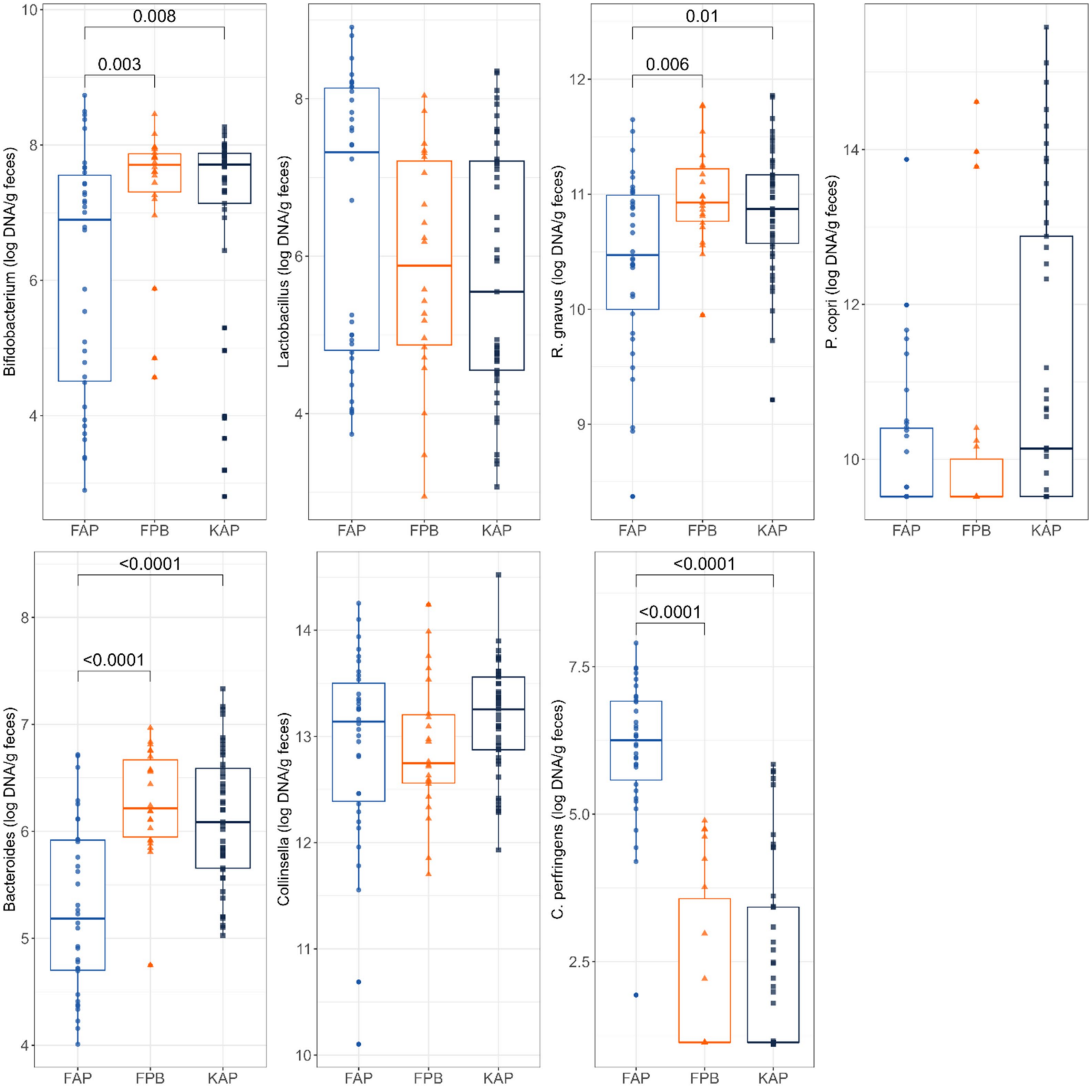
Abundances of fecal *Bifidobacterium*, *Lactobacillus*, *R. gnavus*, *P. copri*, *Bacteroides*, *Collinsella*, and *C. perfringens* (log DNA/g feces) of dogs fed fresh animal protein-based (FAP), fresh plant protein-based (FPB), and kibble animal protein-based (KAP) diets.

Abundances of fecal *Bacteroides*, *Bifidobacterium*, and *C. perfringens* were also impacted by protein source and diet type (Figure 4). Fecal abundance of *Bifidobacterium* was lower in dogs fed FAP diets than those fed FPB diets (*p* = 0.003) and KAP diets (*p* = 0.008). Fecal abundance of *Bacteroides* had a similar pattern, being lower (*p* < 0.0001) in dogs fed FAP diets than those fed FPB and KAP diets. In contrast, fecal *C. perfringens* abundance was higher (*p* < 0.0001) in dogs fed FAP diets than those fed FPB and KAP diets.

**Figure 5:**
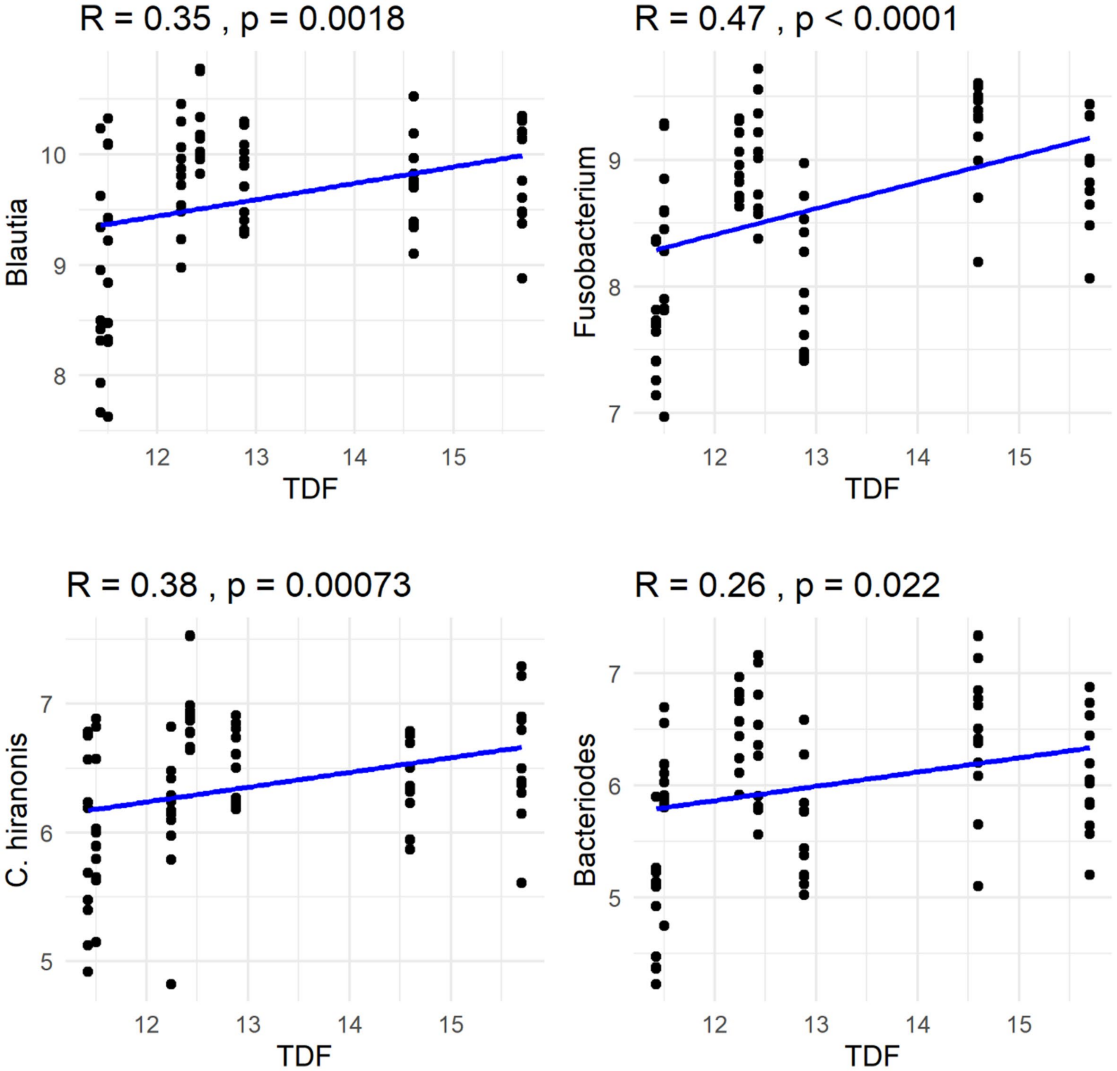
Dietary total dietary fiber (TDF) concentrations were positively correlated with abundances of fecal *Blautia*, *Fusobacterium*, *C. hiranonis*, and *Bacteroides* (log DNA/g feces).

**Figure 6:**
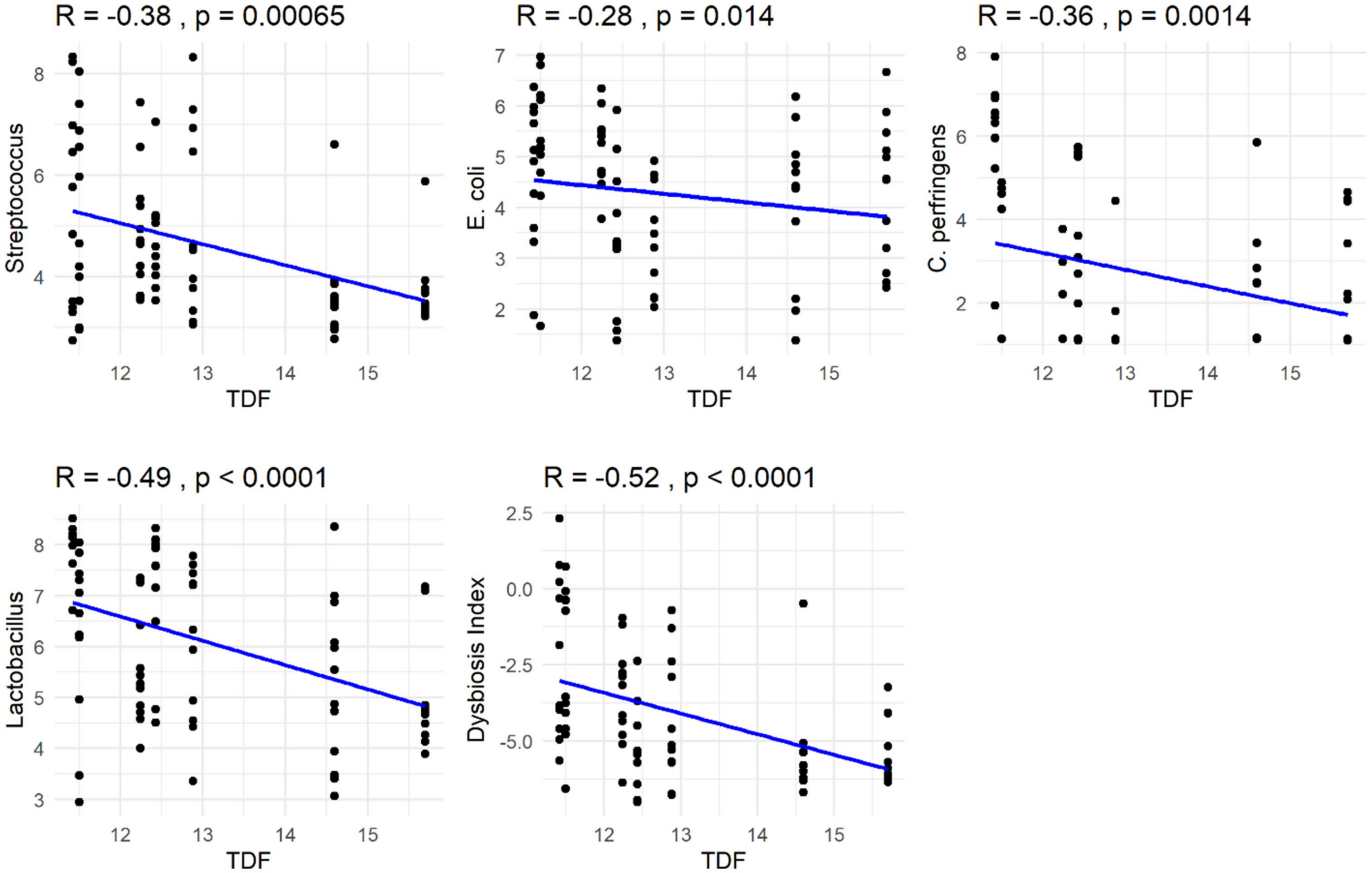
Dietary total dietary fiber (TDF) concentrations were negatively correlated with the abundances of fecal *Streptococcus*, *E. coli*, *C. perfringens*, and *Lactobacillus* (log DNA/g feces), and the dysbiosis index.

**Figure 7:**
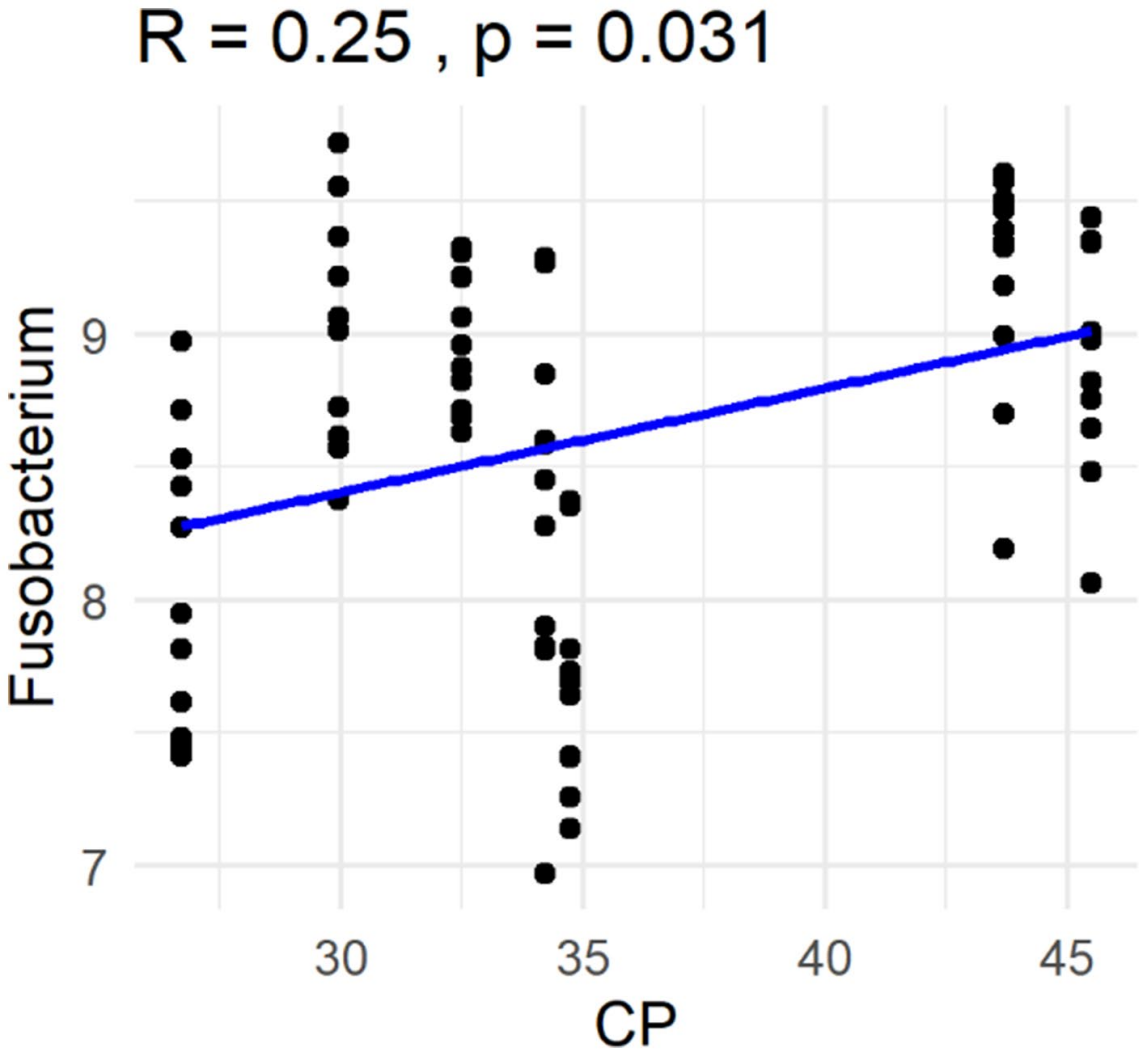
Dietary crude protein (CP) concentrations were positively correlated with the abundance of fecal *Fusobacterium* (log DNA/g feces) and the dysbiosis index.

**Figure 8:**
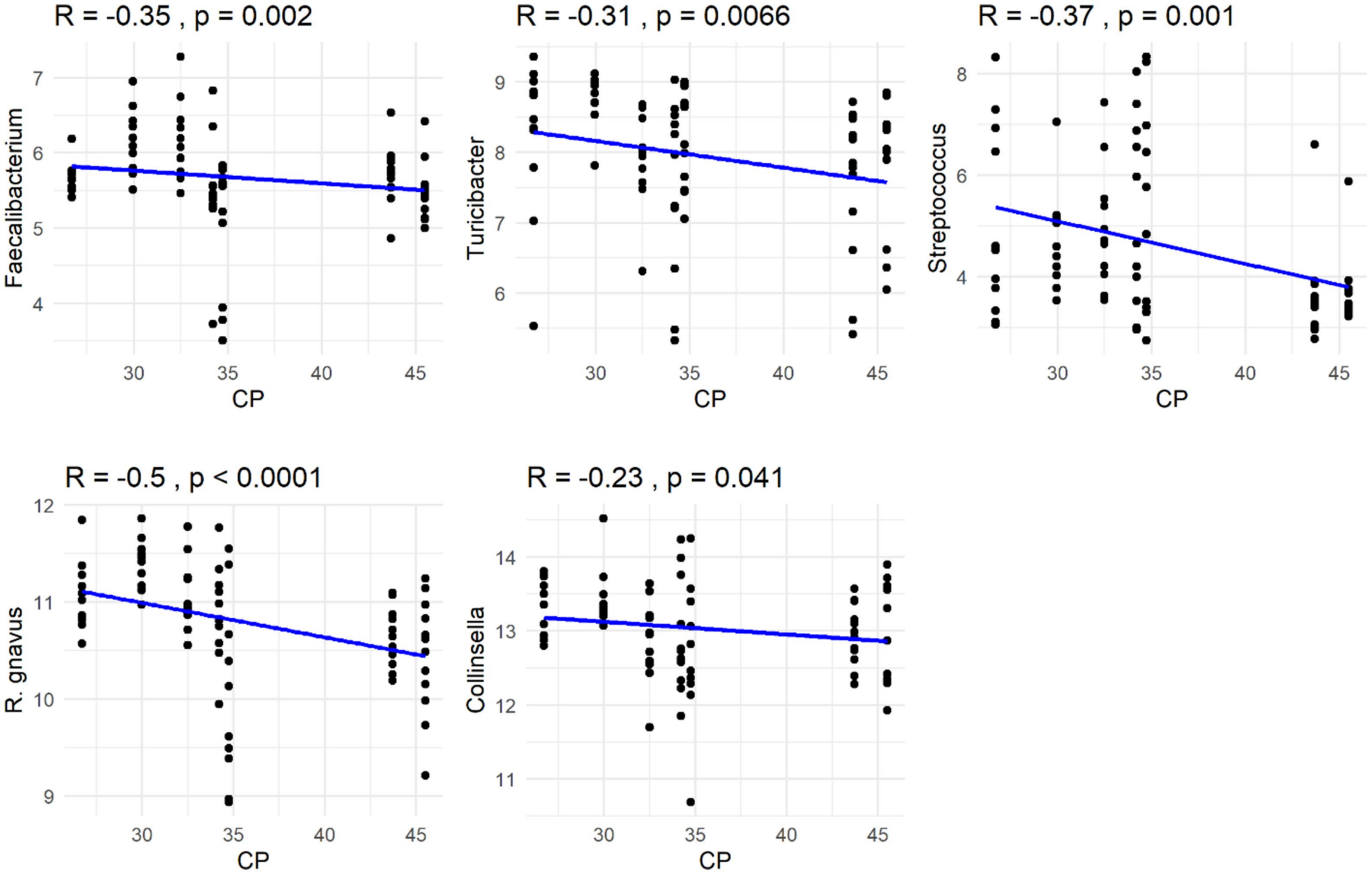
Dietary crude protein (CP) concentrations were negatively correlated with the abundances of fecal *Faecalibacterium*, *Turicibacter*, *Streptococcus*, *R. gnavus*, and *Collinsella* (log DNA/g feces).

**Figure 9:**
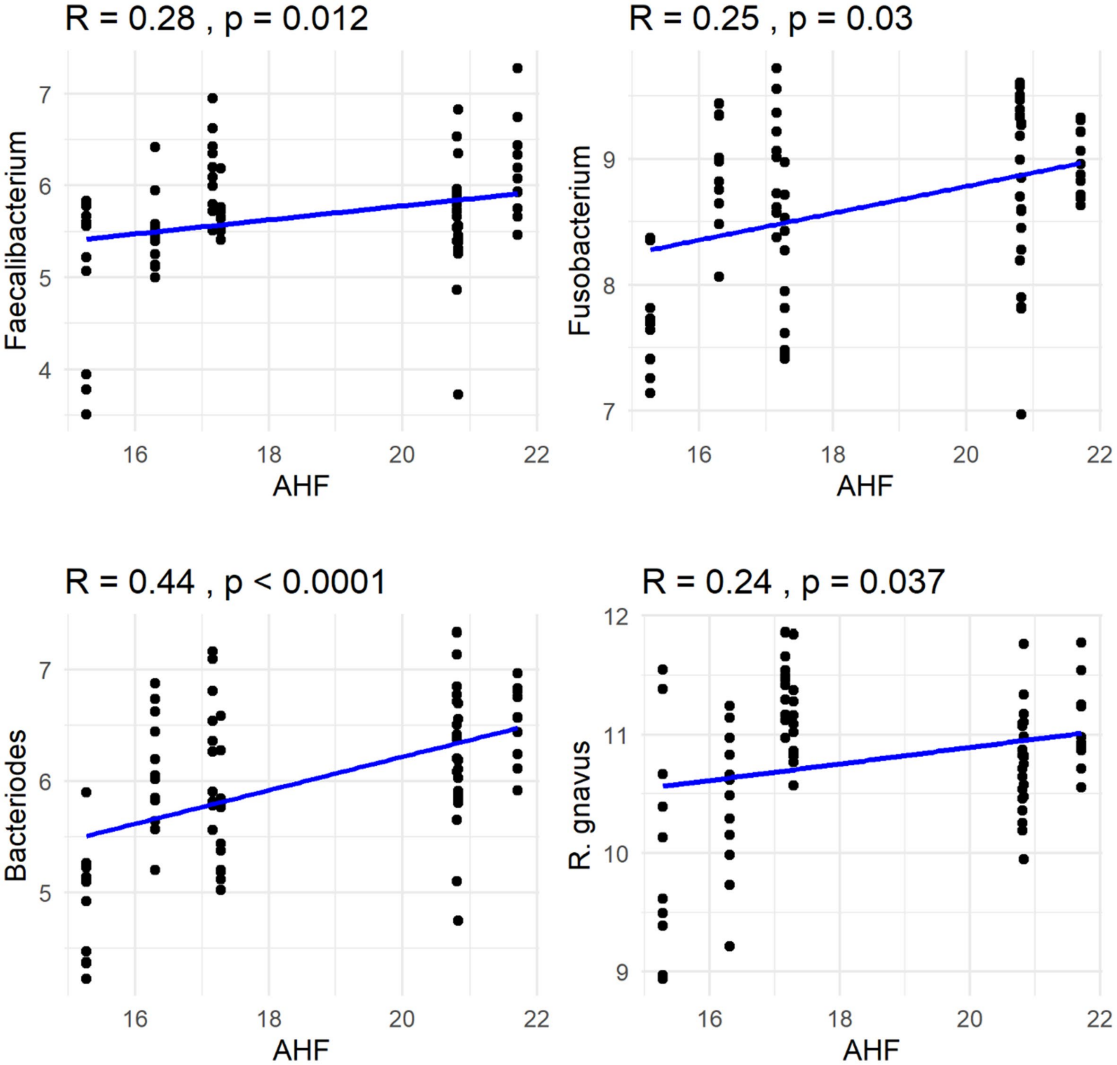
Dietary acid-hydrolyzed fat (AHF) concentrations were positively correlated with abundances of fecal *Faecalibacterium*, *Fusobacterium*, *Bacteroides*, and *R. gnavus* (log DNA/g feces).

**Figure 10:**
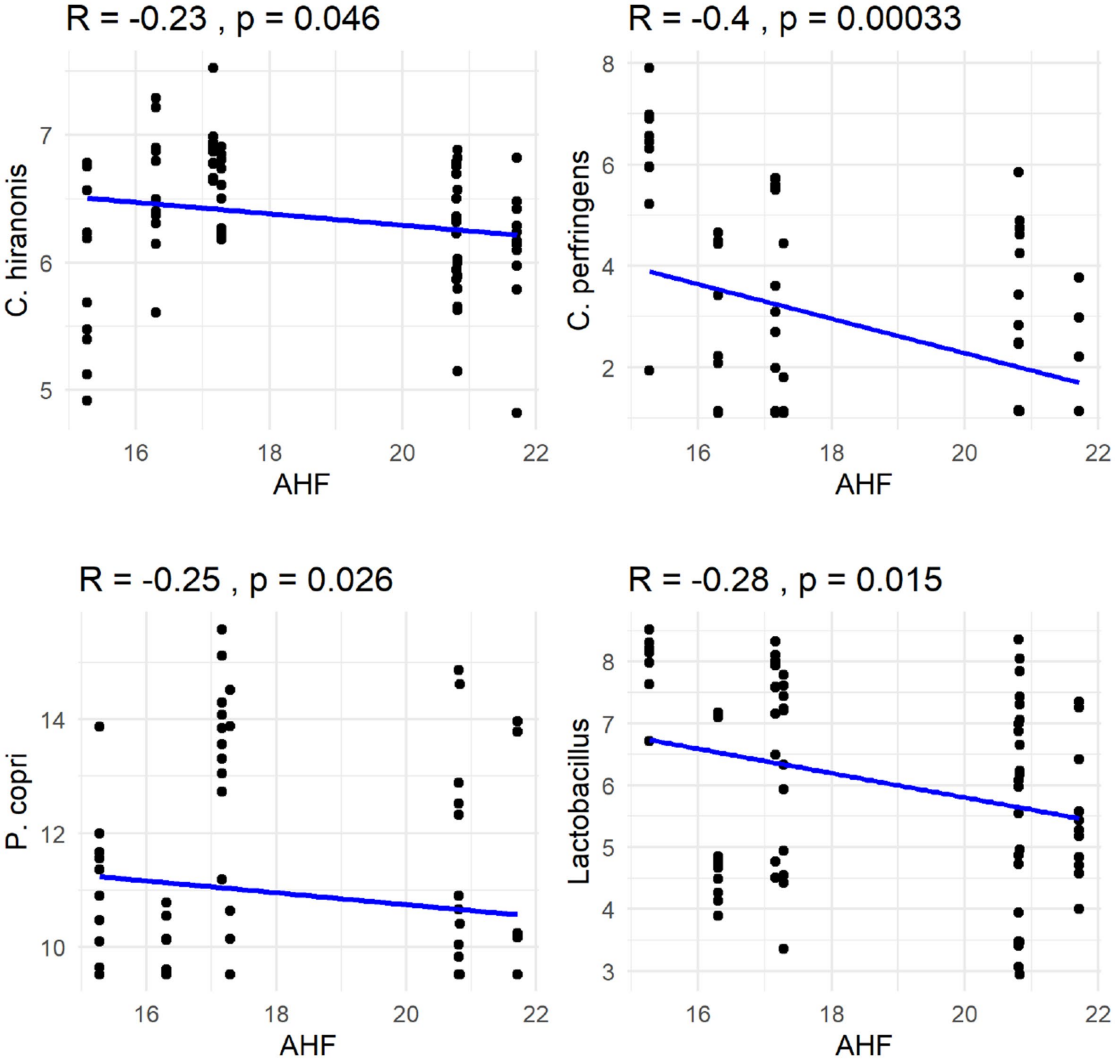
Dietary acid-hydrolyzed fat (AHF) concentrations were negatively correlated with abundances of fecal *C. hiranonis*, *C. perfringens*, *P. copri*, and *Lactobacillus* (log DNA/g feces).

The Spearman correlation coefficients indicated weak positive correlations between dietary total dietary fiber (TDF) concentration and the abundances of fecal *Blautia*, *C. hiranonis*, and *Bacteroides* (R between 0.2 and 0.39), whereas fecal *Fusobacterium* had a moderate positive correlation (R between 0.4 and 0.59) (Figure 5). Additionally, dietary TDF concentration had low negative correlations with fecal *Streptococcus*, *E. coli*, and *C. perfringens* abundances, along with moderate negative correlations with fecal *Lactobacillus* and the DI (Figure 6). For dietary CP concentration, weak positive correlations were observed with *Fusobacterium* abundance (Figure 7). Dietary CP concentration had low negative correlations with fecal *Faecalibacterium*, *Turicibacter*, *Streptococcus*, and *Collinsella* abundances, as well as moderate negative correlations with fecal *R. gnavus* abundance (Figure 8). Dietary acid-hydrolyzed fat (AHF) concentration had weak positive correlations with fecal *Faecalibacterium*, *Fusobacterium*, and *R. gnavus* abundances, but a moderate positive correlation with fecal *Bacteroides* abundance (Figure 9). Finally, dietary AHF concentration had weak negative correlations with fecal *C. hiranonis*, *P. copri*, and *Lactobacillus* abundances, and a moderate correlation with *C. perfringens* abundance (Figure 10).

## 4 Discussion

The DI was trained against the microbiota of dogs with chronic enteropathies, but has also been shown to increase in antibiotic-induced dysbiosis and to correlate well with overall shifts in the canine microbiome across different disease states (23, 30). The major bacterial taxa contributing to intestinal dysbiosis in dogs include *E. coli* (increased), *Streptococcus* (increased), and *Faecalibacterium* (decreased) (24, 31–34). Multiple studies have used the DI as a marker for assessing gastrointestinal health in dogs (24, 35–38). Nevertheless, the impact of diet on the canine microbiome questions the validity of the DI in dogs consuming varied diets. To our knowledge, this is the first study to use qPCR-based DI profiling to compare both animal- and plant-based fresh protein diets with kibble-based animal protein diets in healthy animals.

Recent studies have evaluated the impact that fresh diets have on the canine microbiome, but continued research is necessary to fully comprehend their influence on gastrointestinal and overall host health. One study analyzed fecal samples from 27 dogs fed a home-made high protein and high fat bones and raw food (BARF) and 19 dogs fed commercially available kibble foods using qPCR and the DI (39). Abundances of fecal *E. coli*, *Streptococcus*, and *C. perfringens* and the DI were greater, while abundance of fecal *Faecalibacterium* was lower, in dogs fed BARF than those fed kibble (39). The findings from that study and the current study demonstrate that raw or fresh diets, whether derived from animal- or plant-based proteins, lead to an elevation in fecal *E. coli* and *Streptococcus* abundances. Fresh diets based on animal proteins also resulted in greater fecal *C. perfringens* abundance but lower *Faecalibacterium* abundance. *Faecalibacterium* are SCFA producers, largely use dietary fibers as substrates (29, 40), and are considered to be beneficial to gastrointestinal health, while *C. perfringens*, *E. coli* and *Streptococcus* are considered to be potential pathogenic bacteria (41–43). All animals included in these studies were healthy, however, so the clinical relevance of these shifts is unknown.

High protein intake often elevates fecal protein catabolites (e.g., phenols, indoles, branched-chain fatty acids), reduces SCFA concentrations, and shifts gut microbiota towards potentially harmful genera like *Clostridium*, *E. coli*, and *Streptococcus,* while reducing beneficial bacteria such as *Bifidobacterium* and *Faecalibacterium* (44–46). Such changes may differ based on the source of protein and other nutrients that are also present (e.g., non-digestible oligosaccharides). Fecal *Bifidobacterium* abundance was lower in dogs consuming an animal protein kibble diet than those consuming a fresh plant-based protein diet tested. The opposite was true of fecal *Fusobacterium* abundance, which was lower in dogs consuming an animal protein kibble diet than those consuming a fresh plant-based protein diet (9). Similarly, fecal *Bifidobacterium* abundance was higher in animals consuming fresh animal protein diets than those consuming kibble animal protein diets, while fecal *Bacteroides* abundance was lower in animals consuming fresh animal protein diets than those consuming kibble animal protein diets (8). The results from the current and prior studies suggest that formulas based on animal proteins lead to a reduction in fecal *Bifidobacterium*, *Bacteroides*, and *Fusobacterium* abundances, which are all known SCFA producers (47–50).

The impact of dietary macronutrient content on the gastrointestinal health of animals is intriguing, yet further studies are needed to understand the optimal ratios and their effects. For instance, one study indicated that fecal microbial communities were not different between dogs consuming a plant-based kibble diet or animal protein kibble diet when macronutrient profiles were similar (51). Those results suggest that the significance of ingredients appears to be outweighed by the overall macronutrient content. In healthy adult dogs, consumption of four different types of kibble-based prescription diets – a weight-loss diet (33% protein, 10% fat, 31% fiber), a low-fat diet (24% protein, 8% fat, 10% fiber), a renal diet (15% protein, 20% fat, 8% fiber), and an allergenic diet (19% protein, 18% fat, 6% fiber, all on a dry matter basis)—was associated with changes in microbiome composition (52). Specifically, fecal *Streptococcus* abundances were lower in animals consuming the weight-loss (high fiber and protein) and low-fat diets than those consuming the anallergenic diet, while fecal *Faecalibacterium* abundances were greater in dogs consuming those diets. Additionally, fecal *Fusobacterium* abundance was greater in dogs consuming the weight-loss diet than those consuming the anallergenic diet (52).

In the present study, dietary TDF had a positive correlation with fecal *Fusobacterium* abundance and a negative correlation with fecal *Streptococcus* abundance, which was consistent with previous findings. In a study comparing a low-protein diet (28% CP, 16% fat, 9% TDF in DMB) and a high-protein diet (53% CP, 15% fat, 13% TDF in DMB), fecal *C. hiranonis*, *Streptococcus*, *C. perfringens*, and *R. gnavus* abundances were greater in dogs fed the high-protein diet, while fecal *Fusobacterium*, *Turicibacter*, and *P. copri* abundances were greater in dogs fed the low-protein diet (53). Similar to the previous study, fecal *C. hiranonis* abundance was positively correlated with dietary TDF concentration, while fecal *Turicibacter* abundance was negatively correlated with dietary CP concentration in the current study. *Faecalibacterium* is a SCFA producer and use a variety of fibers as subtrates (29, 40); *C. hiranonis* is capable of dehydroxylating primary bile acids into secondary bile acids, with high-fat diets inducing their proliferation in humans (54, 55). Similar dietary shifts are known to occur with dietary CP, with *Fusobacterium*, a taxa known to ferment amino acids, being positively correlated (56, 57). In the present study, fecal *Bacteroides* abundance was positively correlated with dietary TDF and AHF concentrations. Because *Bacteroides* possess a variety of polysaccharide and protein degradative capabilities (58–61), it is able to adapt to diets differing in nutrient content.

While dietary protein concentration is important, so is the source of protein (e.g., animal or plant). In this study, dogs consuming plant-based fresh and kibble diets had higher fecal *Bifidobacterium*, *R. gnavus*, and *Bacteroides* abundances and lower fecal *C. perfringens* abundance than those consuming a fresh animal-based diet. *R. gnavus* ferments non-digestible carbohydrates to produce acetate, formate, ethanol, propanol, and propionate (62). As noted earlier, *Bacteroides* and *Bifidobacterium* are also SCFA producers (47–50, 64).

This study had some strengths and limitations. One strength included the use of a dog population that was similar in regard to breed (all beagles), sex (all spayed females) and age (all middle aged). Another strength was the fact that all dogs were housed in an experimental facility on the University of Illinois campus, which allowed for strict control of dietary intake, environmental exposures, exercise allowance, light cycle, and other factors that may affect microbiota populations. One limitation was the sample sizes, which were variable (*n* = 10–24/diet) across diets and were modest in some cases. Another limitation was the fact that a single fecal sample was collected from dogs consuming a diet, which did not allow evaluation of microbial stability over time. Lastly, healthy dogs were tested in this study so the clinical relevance of fecal microbial differences were unknown. In addition, while diet categories (FAP, FPB, and KAP) were used to group the foods for analysis, we acknowledge that variability exists within each category, and the use of previously collected data meant that not all diets had complete compositional information available. This reflects the real-world diversity of commercially available diets and is an inherent limitation when evaluating broader dietary categories.

In conclusion, this study compared core bacterial taxa by qPCR and the dysbiosis index and identified several fecal bacterial taxa that differed among dogs fed fresh vs. kibble diets and those based on animal- or plant-based ingredients. In general, dogs fed fresh diets had elevated fecal abundances of potentially pathogenic bacteria (e.g., *E. coli*, *Streptococcus*, *C. perfringens*) and dysbiosis index, and lower fecal abundance of SCFA producers (e.g., *Bacteroides*, *Fusobacterium*). Dogs consuming fresh diets based on animal protein had greater fecal *C. perfringens* abundance and lower fecal *Bacteroides* abundance than those consuming animal kibble diets or plant-based fresh diets. Dietary macronutrient concentrations also affected fecal microbiota, as dietary TDF content was positively correlated with SCFA-producing bacteria (*Blautia*, *Bacteroides*, and *Fusobacterium*), and negatively correlated with potential pathogenic bacteria (*Streptococcus*, *E. coli*, and *C. perfringens*). Dietary protein content was negatively correlated with SCFA-producing bacteria (*Faecalibacterium*, *Turicibacter*, *R. gnavus*, and *Collinsella*). Dietary fat content was positively correlated with SCFA-producing bacteria (*Faecalibacterium*, *Fusobacterium*, *R. gnavus*, and *Bacteroides*), and negatively correlated with potentially pathogenic bacteria (*C. perfringens*). Despite the differences noted in bacterial taxa and dysbiosis across diets, it is important to highlight that average dysbiosis index values remained within the normal range, suggesting that a fresh diet alone does not induce dysbiosis in healthy animals. Further investigations are warranted to fully comprehend the distinct attributes of fresh diet formats and their implications for gastrointestinal health in dogs.

## Data Availability

All relevant data are contained within the article. The original contributions presented in the study are included in the article/Supplementary material. Further inquiries can be directed to the corresponding author.
